# On Prison Discipline
*"Results of the System of Separate Confinement, as administered at the Pentonville Prison." By John T. Burt, B.A., Assistant Chaplain, formerly Chaplain to the Hanwell Lunatic Asylum. 8vo., pp. 287. London: Longmans, 1852.


**Published:** 1853-04-01

**Authors:** 


### Art. III.—ON PRISON DISCIPLINE.*

As the subject of secondary punishment now engages the anxious attention of the legislative and of the executive, any work which professes to place in a true light the effects of any particular system of imprisonment, demands at our hands some consideration. The claim upon our attention becomes stronger, when one qualified by education, by experience, by opportunity, and by integrity, states in plain and unqualified terms the results of his personal observation during a series of years of the working of a System of Imprisonment which has been assailed by popular prejudice, and interfered with by administrative distrust;—a system which, nevertheless, the author affirms (and we are disposed to concur with him), is the best that ever was devised for the *punishment* and for the *reformation* of criminals. The latter object, it must be borne in mind, was a primary object with the founders of the Pentonville prison:—however, it would seem that their successors have abandoned altogether the latter element of prison discipline; and have concentrated all their efforts upon the obtaining only the former—the punitive element.

On the present occasion, we submit to our readers the very important and valuable information which Mr. Burt has to give us upon the past and present condition of the inmates of Pentonville prison; and the consequences which have resulted to the mental health and moral improvement of the prisoners, from an abandonment of the original administration of the Separate system in that establishment. This we shall do by closely following the author in his facts and reasoning.

"The Pentonville prison was erected for the purpose of submitting to actual experiment a new system of prison discipline. That system differed in some of its chief characteristics from all previous systems, and it was subjected to peculiar tests, both from the highly criminal character of the prisoners, and from the long duration of their imprisonment."

"The distinctive characteristic of the discipline was the COMBINATION *of severe punishment with a considerable amount of instruction and other moral influences.* The elements relied upon for severe punishment were, rigid separation, and a protracted term of eighteen months' imprisonment, followed by transportation. The moral or reformatory elements were, frequent visitation by superior officers, a considerable amount of moral and religious instruction, combined with industrial training, and a reasonable prospect of earning an honest livelihood in the colony, upon the sole condition of steady good conduct. At that

* "Results of the System of Separate Confinement, as administered at the Pentonville Prison." By John T. Burt, B.A., Assistant Chaplain, formerly Chaplain to the Hanwell Lunatic Asylum. 8vo., pp. 287. London: Longmans, 1852.

time, these elements of severity and kindness were combined at Pentonville in a higher degree than they have ever been combined in any other prison in Great Britain."—p. 3.

Certain changes have subsequently been introduced; these are thus briefly enumerated:—

"The rigour of the separation has been relaxed; the term of the imprisonment has been reduced; the prospects of the prisoners upon removal have greatly deteriorated; and these and other changes have incidentally, but inevitably, resulted in a decrease of moral instruction. Thus the integrity of the system has in reality been surrendered; a mixed system has superseded it: it is attempted to retain separation without its safeguards, and association is restored for purposes of industry or economy."—p. 4.

The moral results at Pentonville during the early experiment are stated to have been remarkable — the highest degree of satisfaction respecting these was uniformly expressed in the reports of the Commissioners, of the officers, of surgeons of transport ships, and, lastly, of the colonists to whom the prisoners were consigned after their period of probation at Pentonville. The evidence in support of the statement is fully given by Mr. Burt.

These early successful moral results have, by the opponents of the system, been attributed to the careful selection of the early prisoners; but, observes Mr. Burt:—

"The invalidity of this objection to the success of the original discipline is most conclusively established by the fact, that reformation was not by any means limited to those who, according to the only tests of criminality available for the selection, were the least criminal. Prisoners guilty of the graver classes of offences, and previously convicted, succeeded in obtaining conditional pardons and 'tickets-of-leave,' in proportions not greatly varying from those in which the same tokens of reformation were adjudged to others."—p. 29.

The assertion is supported by a table, showing the crimes and classification of the first 1000 prisoners.

The second section of the work brings under notice, "the changes in the system, and their effects." These we shall briefly enumerate, since their description in full would involve the details of the original machinery of the prison. We may draw the reader's attention to the fact, that the separation here spoken of is the separation of the *criminal* from the *criminal*, and not that entire separation and seclusion of the prisoner from all his fellow-creatures which is extensively believed by the uninformed public, and is the notion often fostered at coroners' inquests held by law on the bodies of all British subjects who die in prison.

Mr. Burt thus describes the changes that have been introduced by the present directors:

"1. At first, *separation was rigidly enforced.* The isolation of the criminal from other criminals was the basis of the whole system; therefore it was jealously guarded by all the precautions which long experience had proved indispensable to preclude its evasion. In the construction of the prison, numerous and skilful contrivances were employed in order that the isolation of the cell, of the chapel, of the school, and of the exercising ground, might be preserved inviolate, while, at the same time, the claims of a common humanity were recognised and satisfied."—p. 40.

The author enters into the full relation of the early details, and points out where the discipline has been departed from, in the following and other passages:

"4. The introduction of *the second stage of punishment* has effected a great change in the *prospects* of the prisoners. Originally the prisoner was transported directly from the separate prison to the colony, where he would be removed, as far as practicable, from his former companions in crime, and where he had a reasonable prospect of earning an honest living. At that time, everything was done that could be done, first, by the severity and reformatory influence of the system, to induce him to reform; and then to secure to him the fruits of reformation. Under the existing arrangements, the prisoners are removed from the separate discipline to the hulks or other public works, there to undergo an intermediate imprisonment, in the society of other criminals, often old associates, for terms ranging, according to the length of the sentence, from one year to five, and even to ten years. When that second stage of punishment is past, they are to be transported with tickets of leave; but in the present attitude of the colonies it would be unjustifiable to betray the desponding and confiding convict into any sanguine hopes of well-doing from that remote indulgence. Thus, while at first the prospects of the convict were definite, and, within reasonable limits, encouraging to reformation, now, the hope which formerly sustained him under contrition, and stimulated him to better resolutions, is rendered uncertain and remote.

"Such have been the infringements upon the integrity of the original system. Their expediency upon the grounds of health and economy will be subsequently investigated; our first inquiry is as to the moral results.

"That the introduction of the alterations described has been followed by a great decrease of reformation is a matter of fact, which has been already shown."—p. 44.

The appreciation of the value of the original system, and of the moral injury resulting from these alterations, will necessarily be influenced by the views entertained by different persons with regard to the principles on which the separation should in any case be applied, and upon the estimate they may form of the possibility of prison reformation. It has not, however, been upon any open denial of the importance of this object, that the alterations here alluded to by Mr.

Burt have been made, but upon the assumed strength of certain alleged ill consequences to the mental and bodily health of the prisoners.

Before we proceed to the consideration of the psychological results, we may observe, that Mr. Burt has further made out the strongest possible case in favour of the original Pentonville system, on the grounds of the influence exerted upon the prisoners by the instruction that was imparted, and the hope that was held out to the criminal; and by consequence, an equally strong case appears against the alterations which have decreased the amount of instruction, deprived the criminal of the hope of restoration to respectability and character, and which have exposed him to further contamination by association with other criminals, and so placed reformation at a greater, if not an impossible distance.

We now direct attention to that part of Mr. Burt's work which comes more immediately within the scope of our journal—to the right apprehension of which, however, the preceding extracts are essential; and which, we may add, will be still more intelligible to those who may consult the work itself.

That imprisonment of any kind must to some extent affect the mind is self evident; penal infliction is a disaster to any man; and since in ordinary life "mental disturbance is not unfrequently the consequence of heavy calamities, under any penal system a *degree* of risk to the mind is inevitable." The risk, as the author justly points out, is greater in proportion to the demoralization and criminality resulting out of excess of passion, with defective intelligence; the character generally of the criminal classes.

The psychological question with regard to the separate system is:—"Is this risk excessive?"

The primary requirement of a sound penal discipline, the author argues, is *severity;* this severity, humanity suggests, must be guarded against excess; but as legal enactments must be generally uniform in execution, occasional excess must be inherent in every human system of punishment, and therefore forms no valid objection to the separate system.

The character of the cellular imprisonment, and of the dangers which are attendant thereon, are thus clearly expressed by Mr. Burt.

"Pressure upon the mind under a Cellular System is a necessary concomitant of its characteristic excellence. While corporal punishment degrades a man into a brute, this mode of punishment, combined with instruction, deals with him as a rational being. It deprives him of liberty in all those forms in which it is unsafe that he should be trusted with it; and it induces him to reflect upon his privations, and upon their origin. It appeals, in fact, to his *reason.* When, therefore, the infliction becomes excessively severe, an undue strain upon the power of reason becomes the characteristic danger.

"This admission, however, relates to the *purely penal* element. Under the Separate System, this danger to the mind is counteracted by those other elements which, for want of a more correct term, have been distinguished as *moral* or *reformatory*. The only question is, Whether at Pentonville it was counteracted *completely*?"—p. 91.

Mr. Burt shows that the examination of the mental results of the separate system at Pentonville involves answers to three questions.

First, Whether any of the mental disease under the original system of discipline was the effect of that system, as distinguished from other systems? And if it was so, whether that excess was disproportioned to the comparative efficiency of that discipline? Secondly, What have been the mental results under the altered system, as compared with those under the original system? Thirdly, Whether the earlier or the more protracted period of the term of separate imprisonment has been characterised by the greatest amount of mental disease?

1. Of the mental results under the original system, the reports of the commissioners and of the physician, Dr. Rees, furnish satisfactory evidence.

The proportion of insane during four years, during which the original rigorous discipline remained in force, was as 1·65 in 1000. Allowing for the difficulty of arriving at a comparison of the insanity among the population at large and of Pentonville prison, considering the generally defective character of the statistics of insanity—it still seems that the proportion of insanity at Pentonville, under the original system, was not excessive.

"This conclusion is also carefully deduced in the Report on the Pentonville Prison, by Sir B. C. Brodie and Dr. Fergusson. See also a note to the Sixth Report of the Commissioners, pages 5, 6. It is there shown, 'that in the Society of Friends the cases of insanity occurring annually, between the ages of 20 and 40 years, are in the proportion of 1·5 in 1000.'"

With regard to the comparison that Pentonville would at that time bear with other prisons in this respect, the following note gives information:

"It appears from the gaol return, that during the years 1843—7, in which years the original system was in operation at Pentonville, the annual mean number of criminals brought to trial in England and Wales was 24,785; and that the annual mean number found, or acquitted as insane, was 324. This was in the proportion of 1·3 in 1000. But the number found insane *at their trials* will clearly fall considerably short of the number of cases occurring among the 24,785 criminals *within the year*. The average period which elapsed between the attack and the trial cannot be ascertained; but if, for example sake, we assume it to have been six months, or four months, then the cases of insanity occurring *during an entire year*, would, according to the same rate, be in the proportion of 2·6, or 3·9, in 1000.

"The highest of these proportions is, probably, not much too high. It appears from another table, that the annual mean number of cases of lunacy throughout the prisons of England and Wales, reported *during each of the same years*, was 89·4; and that the average daily population was 14,689. This gives a proportion of 6·3 cases in 1000 prisoners. A very small proportion of these cases may be returned in two consecutive years; and some were insane at the time they were committed to prison. The period during which the attacks occurred, would, in *their cases*, more or less exceed the year. But on the other hand, cases occurring a few weeks before the expiration of the prisoner's sentence, and among prisoners sentenced to only a few weeks' imprisonment, are very often not reported at all. These returns certainly show that the tendency to insanity is very much greater among criminals than it is among the ordinary population."—p. 99.

As the selection of the prisoners sent to Pentonville has been held to be sufficient to explain the earlier results, we quote also Mr. Burt's remarks on that subject.

"But the actual results show that, after all care had been exercised, this selection did not secure a class of prisoners differing much in their mental conditions from the first 469, who were not selected on mental grounds. It has been usual to make inquiry into the previous history of each prisoner *after* his admission. From answers to these inquiries, it was ascertained that among the first 1500 prisoners there were fifty-three who had relatives insane, or who had themselves exhibited indications of unsound mind. Among the first 469 prisoners, who were *not* selected on mental grounds, these unfavourable reports were received in seventeen cases. Among the next 1030, who *were* selected, they were received in thirty-six *cases;* so that the proportion of unfavourable cases was quite as great after the selection as before it. It is also somewhat singular that unfavourable reports were received in the case of both the registered numbers 470 and 471; that is, the first and the second prisoners selected at Millbank."—p. 100.

"If further evidence is required to show how little importance is to be attached to this selection as affecting the actual results, that evidence is found in the fact, that of those fifty-three prisoners not one became the victim of mental disease; on the contrary, with three exceptions, they are reputed to have *improved* under the discipline, and only one was considered to have become decidedly worse. When so large a number of prisoners, known to have been more or less predisposed to insanity, were retained under the most rigorous discipline, for terms in many cases *exceeding eighteen months,* certainly the original system was subjected to a severe test; and when nearly the whole of those prisoners were 'improved,' either mentally or morally, at the expiration of their imprisonment, the result is a very strong proof of the safety of that system as it was then administered."—p. 101.

Mr. Burt brings under prominent notice a form of mental affections described in the prison returns as "delusions." These embrace such persons as, in ordinary life, are frequent enough, but who are not regarded, or at least are not treated, as being insane. They are "eccentric, fanciful, or easily impressed with false conceptions, whether received from others, or generated by a distempered imagination. This class of persons is most numerous amongst the idle, the unmanageable, the reckless habitual drunkards, and those whose intellect is naturally not strong; and those are the characters of which a large portion of the criminal class is composed."

"Under the vigilant supervision exercised at Pentonville these delusions have immediately become matters of record. It would be erroneous, however, to attribute them to the seclusion of the cell, because they first appear, or are first recorded there."

Such cases are met with as frequently in other prisons as in private life. No selection, although it may have prevented the admission of those in whom these delusions were apparent, could have excluded the predisposed to such affections.

The combined testimony of the commissioners, observes Mr. Burt, of the medical commissioner, of the physician, and of the surgeon-superintendent of the transported convicts, all concurred to the effect, that the modified system of separate imprisonment originally inflicted at Pentonville was open to fewer objections, on the score of injury to the mental health, than any other mode of long imprisonment.

The second division of the inquiry into the "mental results" presents us a different and a contrasting picture to what has been drawn by the author in the preceding portion, where it treated of the original discipline.

"The preceding results carry the experiment from the opening of the prison, in 1842, to the end of 1847. In the beginning of 1848, the original term of eighteen months was first reduced to fifteen months, in order to prevent what was thought to have been an excess of mental disease. In this one year, however, there *occurred* five cases of mania, four having occurred before the twelfth month, and the fifth having been produced by a too sudden return to association. Notwithstanding these results, a further reduction of the term took place in 1849, and twelve months was made the maximum period of separation. In this year there occurred four cases of insanity, and a general deterioration in the mental health, which called for special animadversion from the physician. In 1850, there occurred seven cases of insanity. Thus the total number of cases of insanity during three years, under the altered system, was sixteen: the number which had occurred during the preceding four years, while the original system was in full operation, was three; even if the first year is included, the number is six cases in five years. It is clear, therefore, that the amount of insanity has been very much greater in proportion since the original system was disturbed."—p. 110.

"In comparing the results at these two periods, it is unimportant whether we estimate the proportion of the cases to the average daily population, or to the aggregate number of prisoners in the two periods, *compounded* with the duration of the imprisonment undergone by each body of prisoners. The three cases in the four years under the original system, when in full operation, occurred among 1640 prisoners, undergoing *within that period* an average imprisonment of 396 days. The sixteen cases of the last three years, under the altered system, occurred among a population of 2387 prisoners, undergoing, *within that period*, an average imprisonment of 224 days. The difference, therefore, in the proportion of the insane cases at these two periods is as 1 to 8·42; that is, *the insanity, under the altered system has been* EIGHT TIMES *greater than during the four preceding years, when the original system was in* FULL OPERATION. Even if the first year should be included, the proportion under the altered system would be about four times greater than during the first five years of the experiment."—p. 111.

"The general result is sufficient; namely,—that the chief reason alleged for the alteration of the system was a humane desire to prevent mental disease, and that since those alterations have been introduced, there has been a decided and very considerable increase of mental disease."

"A relaxation in the rigour of the separation, and a great reduction in the term of imprisonment, have been followed by a great increase of insanity. The probability, therefore, becomes exceedingly strong, that under the original system the rigour of the separation and the protracted term were *not* the elements upon which the amount of mental disease depended. And this probability is so much the greater, since the effects at the time when the hypothetical cause was in full operation, were scarcely more than were to be accounted for by other causes which are in operation in society at large, without the recognition of any special cause connected with the discipline."—p. 112.

The reduction in the moral elements of the discipline, and in the characters of the criminals, to which Mr. Burt, undoubtedly with justice, attributes these altered results, have already been alluded to.

*Thirdly.*—Whether the more protracted or shorter period of imprisonment has produced the greater amount of insanity will be inferred from what has already been adduced by Mr. Burt, as well as from what follows.

"The deliberate testimony of the commissioners, already adduced, is conclusive evidence to the safety of a protracted term of separation. While in the passages quoted, the Board continued year after year to reiterate their confidence in the original system, the average term of imprisonment was eighteen months, and nearly half the prisoners were detained *beyond* that period, for terms ranging to two years."—p. 125.

"That is to say, so long as actual experiment could have supplied results adverse to the safety of the original term, so long did that experiment fail to supply such results; but, on the contrary, the results obtained were deemed by a Board of great intelligence and great experience, indicative of the exact reverse. Evidence could hardly be stronger."—p. 126.

"By the recent reduction of the term, upon the hypothesis that the separation is attended by increased danger to the mind when prolonged beyond the twelfth month, that hypothesis has been submitted to actual experiment:—and with what results? The reduction was made for the purpose of reducing the mental disease: the more the term has been reduced, the more the insanity has increased. It was reduced first in 1848, and there was an immediate increase; it was reduced again in 1849, and the mental health still further deteriorated; it was practically reduced further in 1850, and the amount of insanity in that one year was as great as in the whole five years under the original system.—p. 128.

"The following Table will show the mental statistics and the population returns for each year since the opening of the prison.

TABLE, *showing the Number of Cases of Mania and of Delusions, and the Numbers of Prisoners admitted and removed, during each Year in* 1843-50.

| | 1843. | 1844. | 1845. | 1846. | 1847. | 1848. | 1849. | 1850. |
|---|---|---|---|---|---|---|---|---|
| Mania . . . . . | 3 | 0 | 1 | 1 | 1 | 5 | 4 | 7 |
| Delusions . . . | 5 | 0 | 2 | 5 | 1 | 2 | 1 | 11 |
| Suicides . . . . | 0 | 0 | 0 | 0 | 0 | 1 | 1 | 1 |
| Admitted . . . . | 525 | 240 | 283 | 243 | 360 | 519 | 599 | 777 |
| Removed . . . . | 24 | 408 | 132 | 386 | 200 | 513 | 621 | 696 |

"From these returns, it is plain that the insanity has invariably increased when a greater number of new prisoners have been admitted, and that it has decreased when the greatest number of old prisoners have been retained in the prison."

The following table further contrasts the results of the two periods.

| Periods. | Average daily Population. | Insane. | Delusions. | Suicides. | Totals. |
|---|---|---|---|---|---|
| 1844, 1845, 1846, 1847, being the period during which the prison contained the greatest number of prisoners undergoing the latter part of the original term of eighteen months . . . . . | 445 | 3 | 10 | 0 | 13 |
| 1843, 1848, 1849, 1850, being the years in which the prison has contained the greatest number of prisoners having undergone less than twelve months of separation . . . . . . . . . . | 486 | 19 | 17 | 3 | 39 |

"These returns are sufficient to show that at Pentonville, under the separate system, the insanity has not increased in consequence of the more protracted term. They prove more; and the more thoroughly the facts are investigated, the more complete the proof becomes, that instead of this hypothetical increase of liability to insanity with the length of the imprisonment, there is a positive *decrease*, and that, when there is any excess of danger to the mind from the discipline, it is during *the earlier, not during the more protracted,* period of imprisonment that the danger is most imminent."

The comparison is followed out by the author thus:—

"The twelfth month is the period which has been assumed as the limit beyond which separation cannot be safely prolonged. It is necessary, therefore, to compare the amount of insanity which has occurred within, with the amount which has occurred beyond that period. From the opening of the prison to the 31st of December, 1850, a period of eight years, there occurred altogether twenty-two cases of insanity: of these there occurred *before* the twelfth month, nineteen; *after* the twelfth month three. During the same period there occurred twenty-six cases of slight mental affection, or delusion: of these there occurred *before* the twelfth month, twenty-two: *after* the twelfth month, four. There have also been three cases of suicide: they have *all* occurred *before* the twelfth month. When these three classes of affections are taken together, there have been in all fifty-one cases; and of these, forty-four have occurred *before*, and seven *after*, the twelfth month.

"But the effect of time upon the development of mental disease at Pentonville, will be more accurately exhibited by distributing these cases under periods of six months.

TABLE, *showing the Periods at which all Cases of Mental Affection have occurred at Pentonville during Eight Years, from the Opening of the Prison on the 22nd December,* 1842, *to the* 31*st December,* 1850.

| Description of Mental Affection. | Six Months and under. | From Six to Twelve Months. | From Twelve to Eighteen Months. | From Eighteen Months to Two Years. |
|---|---|---|---|---|
| Insanity . . . . . . . . . . . | 14 | 5 | 3 | 0 |
| Delusions . . . . . . . . . . . | 13 | 9 | 2 | 2 |
| Suicides . . . . . . . . . . . | 2 | 1 | ... | ... |
| Totals . . . . . . | 29 | 15 | 5 | 2 |

"As the mental cases have been distributed under periods of six months, it will afford a more complete view of the results, if the same distribution is made of the whole of the prisoners whose terms of imprisonment at Pentonville have terminated under the corresponding periods.—p. 133.

TABLE, *showing the Terms of Imprisonment at Pentonville of* 3546 *Prisoners, being the Total Number admitted to the* 31*st December*, 1850, *together with the Mental Cases as reported to that date, distributed under Four Periods of Six Months.*

| Prisoners. | Six Months and under. | From Six to Twelve Months. | From Twelve to Eighteen Months. | From Eighteen Months to Two Years. |
|---|---|---|---|---|
| Removed . . . . . . . . . . . . | 292 | 874 | 1138 | 715 |
| Remaining in the Prison on Dec. 31, 1850 . . . . . . . . . . . | 435 | 83 | 9 | ... |
| Total of Prisoners . . . . . | 727 | 957 | 1147 | 715 |
| Insane . . . . . . . . . . . . . | 14 | 5 | 3 | ... |
| Delusions . . . . . . . . . . . | 13 | 9 | 2 | 2 |
| Suicides . . . . . . . . . . . . | 2 | 1 | ... | ... |
| Total of Mental Cases . . . . | 29 | 15 | 5 | 2 |

TABLE, *showing the Preceding Results distributed under Two Periods of Twelve Months.*

| Class of Prisoners. | Not exceeding Twelve Months. | Exceeding Twelve Months, to the Twenty-fifth Month. |
|---|---|---|
| Prison population . . . . . . . . | 1684 | 1862 |
| Mental cases . . . . . . . . . . | 44 | 7 |

The facts thus adduced are explained by the author in the following paragraph:—

"It is one of the few known laws of mental disease, that periods of transition from one extreme of feeling to its opposite are marked as critical to reason. Men inured to suffering will bear it without much danger. It is the *sudden* inroad of misfortune which either overwhelms the mind, or calls forth too violent an effort of resistance. That excessive effort will be followed by a prostration of the mental energies, and derangement will in some cases ensue, or the mind will be left in the power of slight disturbing causes, until it is rallied under new and invigorating influences. The same law operates even in the case of sudden prosperity. A great and unexpected influx of good fortune will sometimes destroy reason. This is reported to have occurred at the time of the South Sea speculation. A singular example of this law has been supplied at Pentonville, by an occurrence to which I have already adverted. Of the three cases of insanity which have taken place after the twelfth month, one was produced by a too sudden return to association."—p. 136.

It will thus be apparent from the statistics Mr. Burt has thus carefully prepared, and from his whole line of argument, that he arrives very confidently at the conclusion that, "no excess of danger to the mind is to be apprehended from a rigorous adherence to the principle of separation, nor from a protracted period of imprisonment," (p. 138.)

We may add, that the author arrives at very similar results from his investigations into the returns, which state the condition of the bodily health of the prisoners. Our limited space forbids our following the author through the three divisions of his inquiry,—viz., the amount of mortality, the amount of severe sickness, and the general health. We observe, however, that Mr. Burt states the mortality during the period of long imprisonment was 6·15 in the 1000; during the later years of short confinement 7·5 in the 1000; while the mortality of the general population for the ages corresponding with those of the Pentonville prisoners has been ascertained to vary from 9·71 to 12·42 in 1000.

Mr. Burt follows up the preceding parts of his inquiry by a very elaborate elimination of the results of comparisons, of the system at its two periods, and of the separate systems compared with other systems, under its economical aspects, and thence deduces the conclusion, that the efficient application of the original discipline of the cellular system of imprisonment effected a great pecuniary saving to the country, while he states that the present system entails upon the country a yearly charge ranging between 50,000*l.* and 100,000*l.*

It is of course scarcely possible exactly to estimate the relative economy of any one system over another, but it is not difficult to show that the system which most deters from crime, and which effects the greatest amount of moral reformation, must involve also a great saving in the cost of police expenses, and in outlay under the head of criminal jurisprudence. These points, it appears, have been lost sight of under the anxiety to pass as many prisoners as possible through the prison within a given period. Such policy we believe to be in every respect as short sighted as that which follows thereon—viz., the subsequent employment of the convict at an enormous cost, upon public works, to lose in association with other criminals what little reformation may have begun in separation from his fellows in vice, and to be ultimately returned upon society a worse instead of a better member thereof.

Surely our government should pause ere it consents to any plan that involves consequences which experience has shown to have been fraught with incalculable evil and danger in continental countries. A corrupt and fermenting mass of vice cast down into the dregs of society having already enough of grievances, will bring at some future day a full retribution upon those, who for present convenience, have inflicted upon society so grievous an outrage of the laws of humanity and political economy.

We cannot conclude without most strongly recommending Mr. Burt's carefully and well written essay to all interested in psychological and political science. They cannot fail to be greatly enlightened thereby upon a subject, than which few can be named on which there can be found greater misconception and misrepresentation.

---

